# Sleep disturbance partially mediates the association between depression and epilepsy: data from NHANES 2013–2020

**DOI:** 10.1016/j.ebr.2026.100890

**Published:** 2026-07-25

**Authors:** Jiagui Zhong, Qizhen Li, Guoyun Rong, Weixiong Li, Feilin Liang, Yuli Cai, Shumin Bai, Xiaolei Zheng, Wei Li

**Affiliations:** a Dongguan Key Laboratory of Central Nervous System Injury and Repair, The Sixth Affiliated Hospital of Jinan University (Dongguan Eastern Central Hospital), Dongguan 523573, China; b School of Nursing, Guangdong Medical University, Dongguan 523573, China; c Department of Neurosurgery, The Sixth Affiliated Hospital of Jinan University (Dongguan Eastern Central Hospital), Dongguan 523573, China

**Keywords:** Epilepsy, Depression, sleep disturbance, Mediation effect, Heterogeneity

## Abstract

**Objective:**

To examine the association between depression and elevated epilepsy prevalence, and to assess the mediating role of sleep disturbance in this association as well as its heterogeneity across different subpopulations.

**Methods:**

This study included 8223 adults aged ≥18 years from the National Health and Nutrition Examination Survey (NHANES) 2013–2020. Weighted multivariable logistic regression was used to evaluate the association between depression and epilepsy. Mediation analysis was conducted using the PROCESS macro (Model 4), with sleep disturbance as the mediator, and significance was tested via bootstrapping. Interaction terms and stratified analyses were further employed to examine effect modifications by educational level and cardiovascular disease status. All analyses accounted for the complex survey design of NHANES.

**Results:**

Depression was significantly associated with an elevated prevalence of epilepsy. Mediation analysis showed that sleep disturbance partially mediated this association, contributing to 47.34% of the total effect. Stratified analyses indicated that the association varied by educational level and presence of cardiovascular disease.

**Conclusion:**

Based on a nationally representative sample, this study demonstrates that depression is associated with an elevated prevalence of epilepsy both directly and indirectly through sleep disturbance, underscoring the potential importance of mental health and sleep management in epilepsy prevention.

## Highlights

• Depression independently elevates epilepsy risk in U.S. adults.
• Sleep disturbance mediates the depression–epilepsy link.
• Depression–epilepsy association shows marked subgroup heterogeneity.
• Sleep management aids epilepsy primary prevention.

## 1 Introduction

Epilepsy is a prevalent chronic neurological disorder worldwide, characterized by recurrent seizures [1]. It not only severely impairs patients' cognitive function, mental state and quality of life, but also imposes a heavy burden on the public health system. Depression is the most common psychiatric comorbidity in patients with epilepsy, and epidemiological studies have confirmed that the prevalence of depression in the epilepsy population is significantly higher than that in the general population [2]. Moreover, a bidirectional association exists between the two disorders: depression is not merely a psychological consequence after the onset of epilepsy or a side effect of treatment, but also an independent antecedent risk factor for incident epilepsy in adults [3].

Sleep disturbance, a highly prevalent comorbidity of both depression and epilepsy, serves as a potential critical link bridging the two disorders: approximately 80% of patients with major depressive disorder suffer from sleep disorders, and the prevalence of sleep disturbance among epilepsy patients also ranges from 24% to 55% [4], [5]. Basic research has shown that depression and sleep disturbance share biological pathways such as neuronal excitability imbalance, inflammatory activation, and hypothalamic-pituitary-adrenal axis dysfunction [4]. In addition, sleep deprivation can significantly lower the seizure threshold and promote epileptiform discharges, suggesting that sleep disturbance may mediate the association between depression and epilepsy [6].

Although the association between depression and epilepsy has been widely reported, and sleep disturbance is implicated in the pathophysiology of both conditions, the relationships and interactions among these three factors remain poorly understood. Using nationally representative data from 8223 adults in the NHANES 2013–2020, this study aimed to evaluate the association between epilepsy and depression, with a particular focus on the potential mediating role of sleep disturbance.

## 2 Materials and methods

### 2.1 Data source

The data were derived from the National Health and Nutrition Examination Survey (NHANES) database, a nationally representative cross-sectional survey of the U.S. population [7]. This analysis included data from four consecutive 2-year survey cycles spanning 2013–2020. Detailed information regarding the project is accessible on the official NHANES website (https://wwwn.cdc.gov/nchs/nhanes/). The NHANES study protocol was approved by the Institutional Review Board of the National Center for Health Statistics, and all participants provided written informed consent prior to survey enrollment.

### 2.2 Study participants

To examine the association between depression and epilepsy, along with the mediating role of sleep disturbance, this study utilized data from the NHANES 2013–2020. A total of 35,706 participants were initially considered. After excluding individuals under 18 years of age, 21,798 adults remained. We further excluded participants with missing data on key variables, including epilepsy diagnosis, sleep disturbance, and depression. A final sample of 8223 adults was thus included in the statistical analysis (Fig. S1).

### 2.3 Variable definitions

Epilepsy: Epilepsy status was determined through a combination of survey interviews and medication records, following methodologies consistent with prior research [8], [9]. Participants were classified as having epilepsy if they met either of the following criteria: (1)they reported current use of at least one antiepileptic drug primarily indicated for “epilepsy or recurrent seizures” (based on ICD-10 code G40), or (2) self-reported history of recurrent seizures. It should be noted that this epilepsy definition fails to distinguish epileptic seizures from psychogenic non-epileptic, alcohol-withdrawal and other symptomatic provoked seizures, leading to misclassification bias elaborated in the Limitations.

Sleep Disturbance: Information on sleep disturbance was obtained via a self-reported questionnaire item. Participants were asked: “Over the last two weeks, how often have you been bothered by trouble falling or staying asleep, or sleeping too much?” Response options included “several days,” “more than half the days,” or “nearly every day.” Sleep disturbance was defined as a response of “more than half the days” or “nearly every day” [10].

Depression: Depression was assessed using the Patient Health Questionnaire-9 (PHQ-9) from the NHANES questionnaire [11]. This 9-item scale evaluates core depressive symptoms (e.g., depressed mood, sleep disturbance, appetite changes), with a total score ranging from 0 to 27. Using the standard cutoff, a PHQ-9 score ≥ 10 was defined as clinically significant depressive symptoms.

### 2.4 Data collection and variable definitions

Data on sociodemographic characteristics, lifestyle factors, and health conditions were extracted from the NHANES. Variables included: Age, sex, race/ethnicity, educational level, body mass index (BMI), physical activity level, smoking history (defined as having smoked at least 100 cigarettes in one's lifetime), alcohol use (defined as having consumed at least 12 alcoholic drinks in one's lifetime), hypertension, diabetes, hyperlipidemia, cardiovascular disease history, as well as depression and sleep disturbance status [12]

**Operational Definitions:**

Hypertension: Defined by meeting any of the following criteria: self-reported physician diagnosis, measured systolic blood pressure ≥ 140 mmHg, or diastolic blood pressure ≥ 90 mmHg.

Diabetes: Defined by meeting any of the following criteria: self-reported physician diagnosis, current use of insulin or oral hypoglycemic agents, glycated hemoglobin ≥6.5%, or fasting plasma glucose ≥126 mg/dL.

Hyperlipidemia: Defined by meeting any of the following criteria: total cholesterol ≥200 mg/dL, triglycerides ≥150 mg/dL, low-density lipoprotein cholesterol ≥130 mg/dL, high-density lipoprotein cholesterol <40 mg/dL for men or < 50 mg/dL for women, or current use of lipid-lowering medication.

Cardiovascular Disease (CVD): Ascertained based on self-reported physician diagnosis from a standardized questionnaire, encompassing congestive heart failure, coronary heart disease, angina, myocardial infarction, or stroke.

Physical Activity Level: Calculated from the Global Physical Activity Questionnaire by summing the product of metabolic equivalent (MET) values, duration, and frequency for all reported activities to yield total weekly physical activity (MET-min/week). Participants were categorized into low (<500 MET-min/week) and high (≥500 MET-min/week) physical activity groups [12].

Race: Race was categorized into four groups per standard NHANES protocols: non-Hispanic White, non-Hispanic Black, Mexican American, and Others. Remaining Hispanic subgroups were merged into the Others group due to insufficient sample size, which would lead to unstable weighted regression results if analyzed independently. This classification was adopted to match prior NHANES studies.

### 2.5 Statistical analysis

All analyses were performed using R (version 4.4.3) and SPSS 25.0. We accounted for the complex sampling design of the NHANES by incorporating sampling weights, stratification, and clustering variables to generate nationally representative estimates. Continuous and categorical variables were presented as mean (standard error, SE) and weighted percentages, respectively. Group comparisons were performed using independent samples *t*-tests or χ^2^ tests. Weighted multivariable logistic regression was used to evaluate the association between depression and epilepsy, expressed as odds ratios (ORs) with 95% confidence intervals (CI). The mediating role of sleep disturbance was examined using the SPSS PROCESS macro (Model 4) with bootstrapping (5000 resamples). To assess heterogeneity in the associations, interaction terms were included in the models, followed by stratified analyses when a significant interaction (*P* < 0.05) was observed.

This cross-sectional mediation model only estimates correlational rather than causal pathways, since NHANES lacks disease onset timestamps to verify the order of depression, sleep disturbance and epilepsy. We calculated the indirect effect's share of the total association; mediation was significant when the 95% bootstrapped CI for the indirect effect did not contain zero.

## 3 Results

### 3.1 Baseline characteristics of participants

A total of 8223 participants were included in this study. Their baseline characteristics are presented in Table 1. The study population had a mean age of 38.29 ± 0.22 years, with 55.76% being male. A total of 152 participants (1.85%) had epilepsy and 8071 (98.15%) were epilepsy-free. Compared to the non-epilepsy group, the epilepsy group had significant differences in mean age, racial distribution, physical activity level, smoking status, as well as the prevalence of hypertension, diabetes, hyperlipidemia, depression, sleep disturbance, and cardiovascular disease (all *P* < 0.05).  Weighted prevalence data extracted from Table 1 are summarized as follows: overall depression prevalence =5.11%, epilepsy =1.85%; epilepsy affected 17.96% of participants with depression. General population sleep disturbance prevalence was 11.67%, with subgroup rates of 11.33% (no depression/epilepsy), 31.91% (epilepsy only), and elevated levels in comorbid depression-epilepsy individuals.

**Table 1:** Baseline characteristics.

Variable	Total (*n* = 8223)	Non–Epilepsy (*n* = 8071)	Epilepsy (*n* = 152)	Statistic	*P*
Age(years), n(%)	38.29 ± 0.22	38.09 ± 0.23	50.29 ± 1.59	χ^2^ = 90.71	<0.001
˂60	7269 (90.50)	7179 (90.90)	90 (66.60)		
≥60	954 (9.50)	892 (9.10)	62 (33.40)		
Gender, n(%)				χ^2^ = 4.26	0.076
Male	4501 (55.76)	4428 (55.90)	73 (46.99)		
Female	3722 (44.24)	3643 (44.10)	79 (53.01)		
Education, n(%)				χ^2^ = 2.67	0.371
Less than high school	1803 (15.18)	1768 (15.13)	35 (17.87)		
high school or equivalent	1999 (24.62)	1957 (24.55)	42 (28.78)		
college or above	4421 (60.20)	4346 (60.32)	75 (53.36)		
Race, n(%)				χ^2^ = 12.39	0.001
Non-Hispanic Black	1500 (13.16)	1483 (13.27)	17 (6.38)		
Mexican American	2358 (54.93)	2289 (54.70)	69 (68.78)		
Non-Hispanic White	1911 (12.62)	1877 (12.63)	34 (11.81)		
Others	2454 (19.30)	2422 (19.40)	32 (13.03)		
BMI, n(%)				χ^2^ = 4.41	0.097
<30	5476 (66.63)	5393 (66.77)	83 (58.15)		
≥30	2747 (33.37)	2678 (33.23)	69 (41.85)		
physical activity level, n(%)				χ^2^ = 26.33	<0.001
Low physical activity	2418 (25.41)	2345 (25.09)	73 (44.53)		
High physical activity	5805 (74.59)	5726 (74.91)	79 (55.47)		
Smoking, n(%)				χ^2^ = 5.47	0.029
No	5261 (61.33)	5185 (61.49)	76 (51.58)		
Yes	2962 (38.67)	2886 (38.51)	76 (48.42)		
Drinking, n(%)				χ^2^ = 1.95	0.263
No	3023 (31.17)	2956 (31.08)	67 (36.71)		
Yes	5200 (68.83)	5115 (68.92)	85 (63.29)		
Hypertension, n(%)				χ^2^ = 141.27	<0.001
No	6670 (82.95)	6604 (83.59)	66 (44.70)		
Yes	1553 (17.05)	1467 (16.41)	86 (55.30)		
Diabetes, n(%)				χ^2^ = 156.62	<0.001
No	7756 (95.78)	7643 (96.14)	113 (74.24)		
Yes	467 (4.22)	428 (3.86)	39 (25.76)		
Hyperlipidemia, n(%)				χ^2^ = 19.27	<0.001
No	3853 (46.64)	3815 (46.95)	38 (27.90)		
Yes	4370 (53.36)	4256 (53.05)	114 (72.10)		
Depression, n(%)	2.50 (0.05)	2.46 (0.06)	5.18 (0.61)	χ^2^ = 46.51	<0.001
No	7753 (94.89)	7632 (95.11)	121 (82.04)		
Yes	470 (5.11)	439 (4.89)	31 (17.96)		
Sleep disturbance, n(%)				χ^2^ = 54.29	<0.001
No	7260 (88.33)	7149 (88.67)	111 (68.09)		
Yes	963 (11.67)	922 (11.33)	41 (31.91)		
CVD, n(%)				χ^2^ = 790.93	<0.001
No	8015 (97.96)	7912 (98.52)	103 (63.91)		
Yes	208 (2.04)	159 (1.48)	49 (36.09)		

Note: a. Continuous variables are presented as mean ± standard error (SE); categorical variables as weighted percentages (%). b.Group comparisons used chi-square tests; P < 0.05 was statistically significant. c.Depression: PHQ-9 score ≥ 10; sleep disturbance: self-reported sleep problems for >half the days/nearly daily in 2 weeks; epilepsy: ICD-10 G40 antiepileptic drug use or self-reported recurrent seizures. d.BMI:body mass index; CVD:cardiovascular disease; analyses accounted for NHANES 2013–2020 complex sampling design.

### 3.2 Association between depression and epilepsy

Variables with *P* < 0.05 in univariate analyses were included in the multivariable logistic regression model. Sleep disturbance was excluded from this model to avoid overadjustment and potential multicollinearity, which could obscure the primary depression-epilepsy association, and was reserved for subsequent mediation analysis. As shown in Table 2, depression, age, race, physical activity, smoking, hypertension, diabetes, hyperlipidemia, and cardiovascular disease were all significantly associated with epilepsy in the unadjusted model. After adjusting for these covariates, depression remained an independent correlation with elevated epilepsy prevalence (*P* = 0.018, OR = 2.59, 95% CI:1.22–5.52,).

**Table 2:** Factors associated with epilepsy in the study population.

Variables	Univariate analysis					Multivariate analysis					
	β	S.E	t	*P*	OR (95%CI)	β	S.E	t	*P*	OR (95%CI)	
Depression	1.45	0.31	4.61	<0.001	4.25 (2.30–7.87)	0.95	0.39	2.47	0.018	2.59 (1.22–5.52)	
Age， ≥ 60 years	1.61	0.23	7.01	<0.001	5.01 (3.19–7.86)	0.59	0.27	2.19	0.033	1.80 (1.07–3.05)	
Race											
Non-Hispanic Black					1.00 (Reference)					1.00 (Reference)	
Mexican American	0.96	0.34	2.82	0.007	2.62 (1.34–5.10)	0.74	0.36	2.05	0.047	2.09 (1.03–4.22)	
Non-Hispanic White	0.66	0.39	1.71	0.093	1.94 (0.91–4.16)	0.42	0.41	1.03	0.309	1.52 (0.68–3.38)	
Others	0.33	0.39	0.86	0.392	1.40 (0.65–2.98)	0.32	0.40	0.80	0.428	1.38 (0.63–3.05)	
physical activity level，High physical activity	−0.87	0.19	−4.54	<0.001	0.42 (0.29–0.61)	−0.47	0.21	−2.23	0.031	0.62 (0.41–0.94)	
Smoking	0.40	0.19	2.16	0.035	1.50 (1.04–2.16)	−0.14	0.22	−0.65	0.521	0.87 (0.56–1.34)	
Hypertension	1.84	0.21	8.84	<0.001	6.30 (4.19–9.47)	1.01	0.22	4.53	<0.001	2.74 (1.77–4.23)	
Diabetes	2.16	0.27	8.11	<0.001	8.63 (5.13–14.53)	0.97	0.31	3.14	0.003	2.63 (1.44–4.80)	
Hyperlipidemia	0.83	0.26	3.24	0.002	2.29 (1.39–3.77)	0.28	0.31	0.91	0.367	1.32 (0.73–2.40)	
CVD	3.63	0.23	15.92	<0.001	37.66 (24.09–58.86)	2.48	0.30	8.19	<0.001	11.98 (6.61–21.69)	

Note:a. Data presented as β coefficient, S.E., t value, *P* value, OR (95% CI); analyses accounted for NHANES 2013–2020 complex sampling design. b.Non-Hispanic Black race and low physical activity as reference groups; *P* < 0.05 was statistically significant. c.Multivariate model adjusted for all listed variables; sleep disturbance excluded to avoid overadjustment. d.CVD:cardiovascular disease.

### 3.3 Sleep disturbance as a mediator in depression and epilepsy

**Fig. 1:**
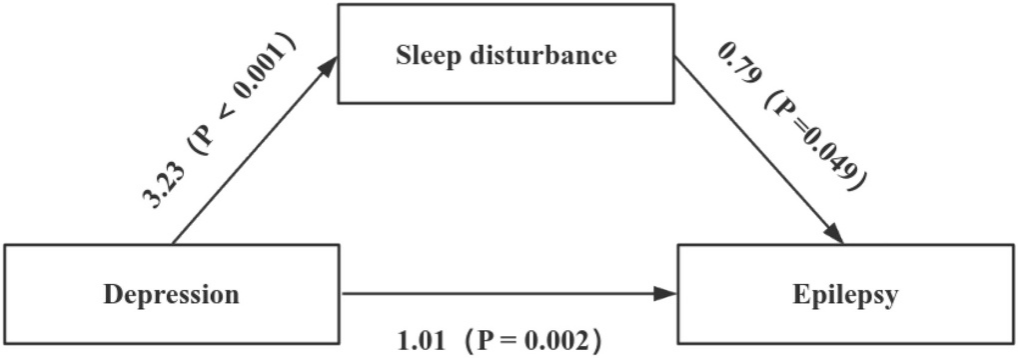
Mediation pathways linking depression and epilepsy through sleep disturbance**.** Depression positively predicted sleep disturbance (β = 3.23, P < 0.001), which in turn was associated with an increased prevalence of epilepsy (β = 0.79, P = 0.049). Analyses used PROCESS Model 4 (5000 bootstraps), accounted for NHANES complex sampling design, and adjusted for covariates; P < 0.05 was statistically significant.

Path analysis (Fig. 1) showed that depression was a significant positive predictor of sleep disturbance (β = 3.23, *P* < 0.001), and sleep disturbance was positively associated with epilepsy (β = 0.79, *P* = 0.049), suggesting sleep disturbance may represent an important mediating pathway linking depression to elevated epilepsy prevalence.

As shown in Table 3, the mediation analysis indicated that sleep disturbance acted as a partial mediator between depression and epilepsy. Specifically, the total effect of depression on epilepsy was 0.0432, which comprised a direct effect of 0.0223 (accounting for 52.66% of the total effect) and an indirect effect through sleep disturbance of 0.0210 (accounting for 47.34%). Bootstrap tests confirmed the statistical significance of both effects, as neither the 95%CI for the direct effect nor that for the indirect effect included zero (both *P* < 0.05). These findings suggest that depression elevates epilepsy prevalence both directly and indirectly via sleep disturbance, indicating that improving sleep may represent a key modifiable target for mitigating the impact of depression on epilepsy.

**Table 3:** Direct and indirect effects of the mediation analysis.

Effect	Efficiency Value	95%CI	P	Effect size
Indirect	0.021	0.0096–0.0379	<0.001	47.34%
Direct	0.0223	0.0003–0.0444	0.040	52.66%
Total	0.0432	0.0193–0.0671	<0.001	100.00%

Note:a. Mediation analysis via PROCESS Model 4 with 5000 bootstrap resamples; *P* < 0.05 considered significant. b. Effect size = percentage of total effect attributable to direct/indirect pathway.

### 3.4 Subgroup and interaction analyses

**Fig. 2:**
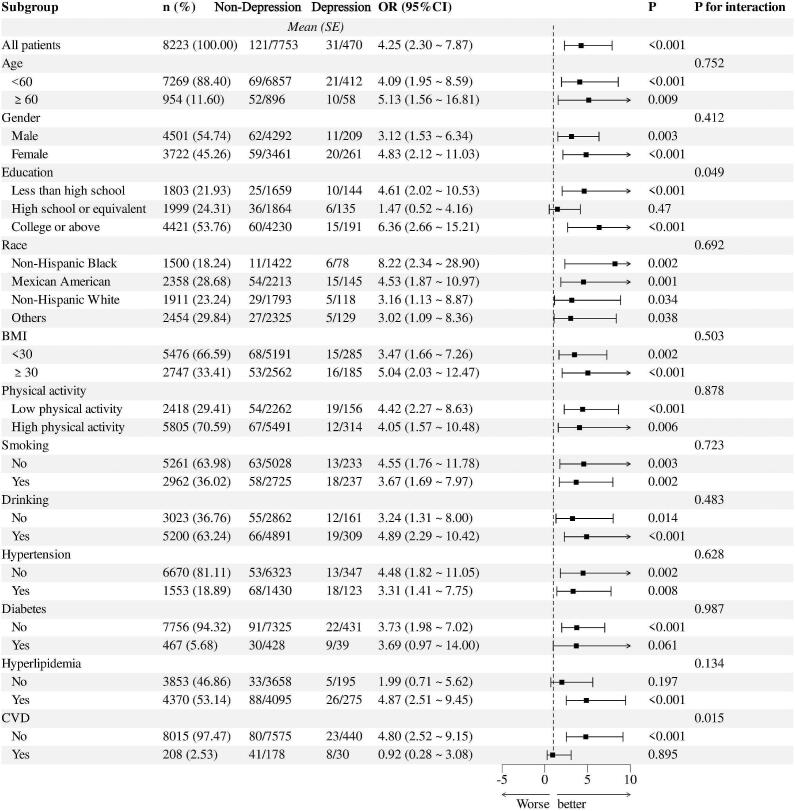
Subgroup analysis of the association between depression and epilepsy**.** A significant positive association between depression and epilepsy was found in the overall population and was consistent across most subgroups; this association was significantly modified by educational level and CVD status.

Subgroup analyses (Fig. 2) revealed a significant positive association between depression and epilepsy in the overall population (OR = 4.25, 95% CI:2.30–7.87, *P* < 0.001). This association remained consistent across the vast majority of subgroups, including those stratified by age, sex, race, body mass index, physical activity, smoking or alcohol consumption status, and chronic comorbidities (all *P* < 0.05).

Interaction analysis indicated that the association was significantly modified by educational level (P for interaction = 0.049) and cardiovascular disease (P for interaction = 0.015). The interaction terms for all other covariates were not statistically significant (all P for interaction >0.05), suggesting that the relationship between depression and epilepsy was generally homogeneous across most population subgroups.

### 3.5 Stratified analysis

To further evaluate the heterogeneity of the association between depression and epilepsy across different educational levels and CVD statuses, stratified analyses were conducted. The results revealed that this cross-sectional association varied notably among these subgroups. A significant positive association between depression and epilepsy was primarily observed in individuals with lower educational attainment and those without CVD (Table 4, Table 5). In contrast, this association was markedly attenuated or became non-significant among participants with higher education or those with comorbid CVD (Tables S1-S3). These findings indicate that both educational level and CVD status serve as significant effect modifiers of the depression-epilepsy association; all subgroup differences reflect observational correlations rather than causal relationships given the cross-sectional study design.

**Table 4:** Epilepsy Prevalence Among Participants with Educational Attainment Less Than High School.

Variables	Univariate analysis						Multivariate analysis				
	β	S.E	t	*P*	OR (95%CI)		β	S.E	t	*P*	OR (95%CI)
Depression	1.53	0.42	3.62	<0.001	4.61 (2.02–10.53)		1.18	0.47	2.52	0.015	3.26 (1.30–8.16)
Age， ≥ 60 years	1.03	0.51	2.00	0.051	2.80 (1.02–7.66)						

Race											
Non-Hispanic Black					1.00 (Reference)						1.00 (Reference)
Mexican American	1.91	0.55	3.48	0.001	6.74 (2.30–19.71)		1.69	0.58	2.92	0.005	5.44 (1.75–16.98)
Non-Hispanic White	1.00	0.71	1.41	0.165	2.71 (0.68–10.87)		0.81	0.73	1.11	0.274	2.24 (0.54–9.35)
Others	1.47	0.68	2.17	0.034	4.36 (1.16–16.47)		1.43	0.66	2.18	0.034	4.20 (1.16–15.24)
physical activity level，High physical activity	−0.43	0.51	−0.85	0.397	0.65 (0.24–1.75)						
Smoking	0.66	0.49	1.35	0.184	1.94 (0.74–5.10)						
Hypertension	1.25	0.39	3.17	0.003	3.47 (1.61–7.51)		0.96	0.48	2.01	0.051	2.61 (1.02–6.67)
Diabetes	1.14	0.42	2.72	0.009	3.14 (1.38–7.14)		0.62	0.52	1.19	0.240	1.86 (0.67–5.15)
Hyperlipidemia	−0.14	0.52	−0.27	0.788	0.87 (0.31–2.41)						
CVD	1.75	0.73	2.38	0.021	5.74 (1.36–24.24)		0.92	1.06	0.87	0.390	2.50 (0.32–19.79)

Note:a. Data presented as β coefficient, S.E., t value, P value, OR (95%CI). b.Non-Hispanic Black race as reference group; P < 0.05 was statistically significant. c.Multivariate model adjusted for significant covariates only; non-significant variables excluded from final model. d.CVD:cardiovascular disease.

**Table 5:** Epilepsy Prevalence Among Participants without Cardiovascular Disease.

Variables	Univariate analysis						Multivariate analysis				
	β	S.E	t	*P*	OR (95%CI)		β	S.E	t	*P*	OR (95%CI)
Depression	1.57	0.33	4.77	<0.001	4.80 (2.52–9.15)		1.53	0.34	4.44	<0.001	4.60 (2.34–9.02)
Age， ≥ 60 years	0.80	0.33	2.43	0.018	2.23 (1.17–4.25)		0.50	0.33	1.52	0.135	1.66 (0.86–3.17)
Race											
Non-Hispanic Black					1.00 (Reference)						1.00 (Reference)
Mexican American	0.86	0.36	2.39	0.020	2.36 (1.17–4.77)		0.88	0.35	2.48	0.017	2.40 (1.20–4.80)
Non-Hispanic White	0.83	0.42	1.97	0.054	2.30 (1.01–5.24)		0.69	0.41	1.67	0.102	1.99 (0.89–4.49)
Others	0.55	0.41	1.35	0.184	1.74 (0.78–3.90)		0.52	0.41	1.28	0.208	1.69 (0.76–3.76)
physical activity level，High physical activity	−0.61	0.28	−2.20	0.032	0.54 (0.32–0.93)		−0.53	0.28	−1.91	0.062	0.59 (0.34–1.01)
Smoking	0.21	0.25	0.85	0.399	1.23 (0.76–1.99)						
Hypertesion	1.04	0.27	3.80	<0.001	2.83 (1.66–4.84)		0.81	0.27	2.94	0.005	2.24 (1.31–3.83)
Diabetes	1.16	0.27	4.24	<0.001	3.19 (1.87–5.45)		0.83	0.32	2.62	0.012	2.30 (1.23–4.28)
Hyperlipidemia	0.19	0.29	0.65	0.521	1.21 (0.68–2.12)						

Note:a. Data presented as β coefficient, S.E., t value, P value, OR (95% CI). b.Non-Hispanic Black race as reference group; P < 0.05 was statistically significant. c.Multivariate model adjusted for significant covariates only; non-significant variables excluded from final model. d.CVD:cardiovascular disease.

## 4 Discussion

This study, utilizing a nationally representative sample of U.S. adults, demonstrates a significant positive association between depression and epilepsy and further identifies sleep disturbance as a partial mediator in this relationship. These findings extend prior epidemiological evidence on the bidirectional link between depression and epilepsy by suggesting that depression is not merely a comorbidity but may also contribute to epileptogenesis through potentially modifiable pathways such as sleep disruption.

Previous studies indicate that depression is the most common psychiatric comorbidity in patients with epilepsy, a relationship mediated by a combination of neurobiological, psychosocial, and iatrogenic factors [13], [14]. Our data confirm that depression increases the prevalence of epilepsy in adulthood. Case-control studies further suggest that individuals with depression have a 2- to 7-fold higher prevalence of epilepsy compared to the general population [15]. Mechanistically, depression-accociated hyperactivity of the hypothalamic-pituitary-adrenal axis, chronic low-grade inflammation, and dysregulation in neurotransmitters (glutamate and GABA) may lower the seizure threshold [16], [17], [18]. In addition, our findings indicate that depression may also indirectly correlate with elevated epilepsy prevalence through sleep disturbance. Sleep disorders, such as chronic sleep deprivation or fragmentation, can increase cortical excitability and promote epileptiform discharges, thereby acting as a bridge in the pathological cascade from depression to epilepsy [6]. Recent evidence suggests that sleep drive, rather than sleep duration, is the main contributor to the worsening of seizures after sleep loss [19]. Our mediation analysis quantifies this pathway and provides empirical support for future preventive research targeting sleep interventions. Future translational research could validate these putative biological pathways via prospective biomarker cohort studies, neuroimaging and EEG assessments of neuronal excitability, preclinical animal models, and clinical sleep-modifying intervention trials to clarify the causal physiological links between depressive symptoms and epilepsy.

Importantly, we also observed notable heterogeneity in the depression-epilepsy association across different educational levels and cardiovascular disease status. The association was stronger among individuals with lower educational attainment and those without cardiovascular disease. This pattern likely reflects the combined influence of socioeconomic status, health literacy, healthcare access, and health behaviors (including sleep hygiene), with these factors amplifying the pathophysiological impact of depression [20], [21], [22].Recognizing this heterogeneity is important for developing stratified prevention strategies.

Clinically, these results position individuals with depression as a higher-risk group for epilepsy, warranting routine sleep disturbance screening and management. Interventions for depression might consider incorporating sleep-directed therapies such as cognitive behavioral therapy for insomnia alongside antidepressant treatment, given that sleep disturbance is a core symptom of depression that may not be fully resolved by pharmacotherapy alone. However, whether targeting this pathway reduces seizure risk remains to be tested in future prospective studies. From a public-health perspective, integrating mental and sleep health metrics into primary prevention and risk-stratification frameworks would represent an upstream shift in epilepsy prevention.

However, the cross-sectional design precludes definitive causal inferences. Future prospective or interventional studies are needed to establish causality and validate the pathways. Concurrently, mechanistic studies utilizing neuroimaging, electrophysiology, and molecular biology are essential to uncover the biological basis of these associations and inform targeted prevention strategies. Second, our epilepsy definition without EEG or imaging verification may misclassify psychogenic and provoked seizures as true epilepsy and overestimate prevalence. This bias exerts two potential impacts on our mediation results: similar mental health burden in misclassified subjects leaves our mediation effect unchanged, while weaker correlations would slightly underestimate our total and indirect effects. We have noted this inherent NHANES limitation and emphasized the need for prospective clinical validation. Third, the cross-sectional nature of NHANES prevents confirmation of the temporal sequence between depression, sleep disturbance and epilepsy, limiting causal inference.

## 5 Conclusion

This study demonstrates that depression is associated with a substantial risk of epilepsy, with this association partially mediated by sleep disturbance. This association varies by sociodemographic and clinical characteristics. Integrated sleep problem management in patients with depression represents a novel avenue for epilepsy prevention, a direction that warrants verification in future prospective studies.

## CRediT authorship contribution statement

**J. Zhong:** Writing – original draft, Methodology, Data curation, Visualization, Conceptualization, Funding acquisition. **Q. Li:** Writing – review & editing, Writing – original draft, Methodology, Data curation, Visualization, Conceptualization. **G. Rong** and **W. Li:** Resources, Validation, Supervision. **F. Liang**, **Y. Cai** and **S. Bai:** Writing – review & editing, Validation, Supervision, Project administration. **X. Zheng** and **W. Li:** Writing – review & editing, Validation, Supervision, Project administration, Methodology, Conceptualization.

## Consent for publication

Not applicable.

## Ethical statement

This study utilized exclusively de-identified, aggregated data from the National Health and Nutrition Examination Survey. The study was conducted in accordance with the Declaration of Helsinki, and no additional ethical approval or individual patient consent was required for this analysis.

## Ethics approval and consent to participate

This study utilized exclusively de-identified, aggregated data from the National Health and Nutrition Examination Survey. The study was conducted in accordance with the Declaration of Helsinki, and no additional ethical approval or individual patient consent was required for this analysis.

## Funding

This study was supported by the Medical Scientific Research Foundation of Guangdong Province, China (Number: 20241114165757452)

## Declaration of competing interest

The authors declare that they have no known competing financial interests or personal relationships that could have appeared to influence the work reported in this paper.

## Data Availability

Data used in this study were obtained from the National Health and Nutrition Examination Survey (https://wwwn.cdc.gov/nchs/nhanes/).
