# Supplementary material for: Sleep disturbance partially mediates the association between depression and epilepsy: data from NHANES 2013–2020
Source: Epilepsy Behav Rep. 2026 Jul 25;35:100890. doi: 10.1016/j.ebr.2026.100890 (PMC13463980; doi:10.1016/j.ebr.2026.100890)
Supplement: Supplementary file 1 [file mmc1.docx]

**Sleep Disturbance Partially Mediates the Association Between Depression and Epilepsy: data from NHANES 2013–2020**

Manuscript ID: EBR 100890

**Supplement figure and table:**

**Fig S1. Flowchart of participant inclusion.** Participants were enrolled from the NHANES (2013–20200. A total of 8,223 eligible adults were included in the final analysis after applying inclusion and exclusion criteria.

**Table S1. Risk of Epilepsy Among Participants with Educational Ettainment of High School or equivalent**

| Variables | Univariate analysis | | | | |  | Multivariate analysis | | | | |
| --- | --- | --- | --- | --- | --- | --- | --- | --- | --- | --- | --- |
|  | β | S.E | t | *P* | OR (95%CI) |  | β | S.E | t | *P* | OR (95%CI) |
| Depression | 0.39 | 0.53 | 0.73 | 0.470 | 1.47 (0.52 ~ 4.16) |  |  |  |  |  |  |
| Age，≥60 years | 1.19 | 0.39 | 3.01 | 0.004 | 3.28 (1.51 ~ 7.10) |  | 0.21 | 0.42 | 0.50 | 0.616 | 1.24 (0.54 ~ 2.85) |
| Race |  |  |  |  |  |  |  |  |  |  |  |
| Non-Hispanic Black |  |  |  |  | 1.00 (Reference) |  |  |  |  |  | 1.00 (Reference) |
| Mexican American | 2.18 | 0.78 | 2.80 | 0.007 | 8.87 (1.92 ~ 40.83) |  | 1.81 | 0.71 | 2.54 | 0.014 | 6.14 (1.51 ~ 24.89) |
| Non-Hispanic White | 2.01 | 0.80 | 2.50 | 0.015 | 7.43 (1.55 ~ 35.68) |  | 1.56 | 0.79 | 1.99 | 0.053 | 4.77 (1.02 ~ 22.23) |
| Others | 2.24 | 0.83 | 2.70 | 0.009 | 9.43 (1.85 ~ 47.94) |  | 2.30 | 0.78 | 2.94 | 0.005 | 10.02 (2.15 ~ 46.65) |
| physical activity level，High physical activity | -0.46 | 0.33 | -1.40 | 0.167 | 0.63 (0.33 ~ 1.20) |  |  |  |  |  |  |
| Smoking | 0.20 | 0.35 | 0.58 | 0.567 | 1.22 (0.62 ~ 2.42) |  |  |  |  |  |  |
| Hypertension | 1.82 | 0.44 | 4.11 | <0.001 | 6.16 (2.59 ~ 14.66) |  | 0.89 | 0.37 | 2.38 | 0.022 | 2.43 (1.17 ~ 5.05) |
| Diabetes | 2.70 | 0.55 | 4.91 | <0.001 | 14.86 (5.06 ~ 43.63) |  | 1.15 | 0.56 | 2.05 | 0.046 | 3.17 (1.05 ~ 9.56) |
| Hyperlipidemia | 0.89 | 0.50 | 1.78 | 0.081 | 2.43 (0.91 ~ 6.49) |  |  |  |  |  |  |
| CVD | 3.91 | 0.46 | 8.51 | <0.001 | 49.94 (20.30 ~ 122.87) |  | 3.00 | 0.34 | 8.70 | <0.001 | 20.00 (10.19 ~ 39.27) |

Notes: Data are presented as β coefficient, S.E., P value, and OR (95% CI). Analyses accounted for NHANES 2013–2020 complex sampling design and were adjusted for covariates. P<0.05 was considered statistically significant. OR：odds ratio; CI：confidence interval; S.E.：standard error; CVD：cardiovascular disease.

**Table S2. Risk of Epilepsy Among Participants with Educational Attainment of College or above**

| Variables | Univariate analysis | | | | |  | Multivariate analysis | | | | |
| --- | --- | --- | --- | --- | --- | --- | --- | --- | --- | --- | --- |
|  | β | S.E | t | *P* | OR (95%CI) |  | β | S.E | t | *P* | OR (95%CI) |
| Depression | 1.85 | 0.44 | 4.16 | <0.001 | 6.36 (2.66 ~ 15.21) |  | 1.13 | 0.62 | 1.82 | 0.075 | 3.09 (0.92 ~ 10.40) |
| Age，≥60 years | 1.98 | 0.30 | 6.61 | <0.001 | 7.24 (4.03 ~ 13.01) |  | 0.79 | 0.39 | 2.02 | 0.049 | 2.19 (1.03 ~ 4.70) |
| Race |  |  |  |  |  |  |  |  |  |  |  |
| Non-Hispanic Black |  |  |  |  | 1.00 (Reference) |  |  |  |  |  | 1.00 (Reference) |
| Mexican American | 0.19 | 0.46 | 0.40 | 0.688 | 1.20 (0.49 ~ 2.97) |  | -0.37 | 0.43 | -0.85 | 0.399 | 0.69 (0.30 ~ 1.61) |
| Non-Hispanic White | -0.11 | 0.53 | -0.20 | 0.842 | 0.90 (0.32 ~ 2.55) |  | -0.42 | 0.54 | -0.78 | 0.439 | 0.66 (0.23 ~ 1.88) |
| Others | -1.50 | 0.58 | -2.59 | 0.012 | 0.22 (0.07 ~ 0.69) |  | -1.76 | 0.56 | -3.12 | 0.003 | 0.17 (0.06 ~ 0.52) |
| physical activity level，High physical activity | -1.22 | 0.24 | -5.12 | <0.001 | 0.30 (0.19 ~ 0.47) |  | -0.75 | 0.26 | -2.85 | 0.006 | 0.47 (0.28 ~ 0.79) |
| Smoking | 0.37 | 0.24 | 1.56 | 0.125 | 1.44 (0.91 ~ 2.29) |  |  |  |  |  |  |
| Hypertension | 2.04 | 0.24 | 8.63 | <0.001 | 7.67 (4.83 ~ 12.19) |  | 1.14 | 0.27 | 4.16 | <0.001 | 3.11 (1.82 ~ 5.31) |
| Diabetes | 2.15 | 0.35 | 6.07 | <0.001 | 8.57 (4.28 ~ 17.16) |  | 0.73 | 0.46 | 1.58 | 0.121 | 2.09 (0.84 ~ 5.18) |
| Hyperlipidemia | 1.14 | 0.37 | 3.08 | 0.003 | 3.14 (1.52 ~ 6.49) |  | 0.53 | 0.44 | 1.22 | 0.230 | 1.71 (0.72 ~ 4.04) |
| CVD | 4.01 | 0.32 | 12.33 | <0.001 | 54.90 (29.04 ~ 103.77) |  | 2.69 | 0.40 | 6.71 | <0.001 | 14.71 (6.70 ~ 32.26) |

Notes: Data are presented as β coefficient, S.E., P value, and OR (95% CI). Analyses accounted for NHANES 2013–2020 complex sampling design and were adjusted for covariates. P<0.05 was considered statistically significant. OR：odds ratio; CI：confidence interval; S.E.：standard error; CVD：cardiovascular disease.

**Table S3. Risk of Epilepsy Among Participants with Cardiovascular Disease**

| Variables | Univariate analysis | | | | |  | Multivariate analysis | | | | |
| --- | --- | --- | --- | --- | --- | --- | --- | --- | --- | --- | --- |
|  | β | S.E | t | *P* | OR (95%CI) |  | β | S.E | t | *P* | OR (95%CI) |
| Depression | -0.08 | 0.62 | -0.13 | 0.895 | 0.92 (0.28 ~ 3.08) |  |  |  |  |  |  |
| Age，≥60 years | 1.20 | 0.53 | 2.25 | 0.031 | 3.31 (1.17 ~ 9.37) |  | 1.28 | 0.51 | 2.51 | 0.018 | 3.58 (1.32 ~ 9.71) |
| Race |  |  |  |  |  |  |  |  |  |  |  |
| Non-Hispanic Black |  |  |  |  | 1.00 (Reference) |  |  |  |  |  |  |
| Mexican American | 0.53 | 0.77 | 0.68 | 0.500 | 1.69 (0.37 ~ 7.70) |  |  |  |  |  |  |
| Non-Hispanic White | -0.61 | 0.78 | -0.79 | 0.438 | 0.54 (0.12 ~ 2.48) |  |  |  |  |  |  |
| Others | -0.75 | 0.95 | -0.78 | 0.439 | 0.47 (0.07 ~ 3.07) |  |  |  |  |  |  |
| physical activity level，High physical activity | -0.83 | 0.40 | -2.06 | 0.047 | 0.44 (0.20 ~ 0.96) |  | -0.22 | 0.41 | -0.53 | 0.598 | 0.80 (0.36 ~ 1.80) |
| Smoking | -0.25 | 0.41 | -0.62 | 0.542 | 0.78 (0.35 ~ 1.72) |  |  |  |  |  |  |
| Hypertension | 2.41 | 0.47 | 5.09 | <0.001 | 11.14 (4.41 ~ 28.19) |  | 1.86 | 0.48 | 3.85 | <0.001 | 6.42 (2.49 ~ 16.53) |
| Diabetes | 1.48 | 0.54 | 2.75 | 0.010 | 4.38 (1.53 ~ 12.57) |  | 1.32 | 0.63 | 2.11 | 0.044 | 3.75 (1.10 ~ 12.82) |
| Hyperlipidemia | 3.18 | 0.93 | 3.43 | 0.002 | 24.05 (3.91 ~ 148.13) |  | 3.32 | 1.05 | 3.16 | 0.004 | 27.67 (3.53 ~ 216.97) |

Notes: Data are presented as β coefficient, S.E., P value, and OR (95% CI). Analyses accounted for NHANES 2013–2020 complex sampling design and were adjusted for covariates. P<0.05 was considered statistically significant. OR：odds ratio; CI：confidence interval; S.E.：standard error;.
